# The relation between socioeconomic status and short-term mortality after acute myocardial infarction persists in the elderly: results from a nationwide study

**DOI:** 10.1007/s10654-012-9700-z

**Published:** 2012-06-05

**Authors:** Aloysia A. M. van Oeffelen, Charles Agyemang, Michiel L. Bots, Karien Stronks, Carla Koopman, Lenie van Rossem, Ilonca Vaartjes

**Affiliations:** 1 Julius Center for Health Sciences and Primary Care, University Medical Center Utrecht, 3508 GA Utrecht, The Netherlands; 2 Department of Public Health, Academic Medical Center Amsterdam, Amsterdam, The Netherlands

**Keywords:** Myocardial infarction, Short-term mortality, Socioeconomic status, Income, Elderly

## Abstract

We assessed whether the previously observed relationship between socioeconomic status (SES) and short-term mortality (pre-hospital mortality and 28-day case-fatality) after a first acute myocardial infarction (AMI) in persons <75 years, are also observed in the elderly (i.e. ≥75 years), and whether these relationships vary by sex. A nationwide register based cohort study was conducted. Between January 1st 1998 and December 31st 2007, 76,351 first AMI patients were identified, of whom 60,498 (79.2 %) were hospitalized. Logistic regression analyses were performed to measure SES differences in pre-hospital mortality after a first AMI and 28-day case-fatality after a first AMI hospitalization. All analyses were stratified by sex and age group (<55, 55–64, 65–74, 75–84, ≥85), and adjusted for age, ethnic origin, marital status, and degree of urbanization. There was an inverse relation between SES and pre-hospital mortality in both sexes. There was also an inverse relation between SES and 28-day case-fatality after hospitalization, but only in men. Compared to elderly men with the highest SES, elderly men with the lowest SES had a higher pre-hospital mortality in both 75–84 year-olds (OR = 1.26; 95 % CI 1.09–1.47) and ≥85 year-olds (OR = 1.26; 1.00–1.58), and a higher 28-day case-fatality in both 75–84 year-olds (OR = 1.26; 1.06–1.50) and ≥85 year-olds (OR = 1.36; 0.99–1.85). Compared to elderly women with the highest SES, elderly women with the lowest SES had a higher pre-hospital mortality in ≥85 year-olds (OR = 1.20; 0.99–1.46). To conclude, in men there are SES inequalities in both pre-hospital mortality and case-fatality after a first AMI, in women these SES inequalities are only shown in pre-hospital mortality. The inequalities persist in the elderly (≥75 years of age). Clinicians and policymakers need to be more vigilant on the population with a low SES background, including the elderly.

## Introduction

In the last decades the mortality after a cardiovascular event has steadily declined in Western societies [1, 2], though a socioeconomic status (SES) gradient still persists [3]. Acute myocardial infarction (AMI) is one of the major cardiovascular diseases with a SES gradient in short-term mortality as well as long-term mortality [4]. Short-term mortality after an AMI comprises of pre-hospital mortality and case-fatality. Pre-hospital mortality is defined as dying after a first AMI attack before hospitalization; case-fatality is defined as dying within 28 days after the first AMI hospitalization. Pre-hospital mortality has often shown to be stronger related to SES than case-fatality [5–8]. This may be due to the clear relationship between SES and an unfavorable risk factor profile (e.g. smoking, unhealthy diet, inactivity, stress) [9], which is more influential on pre-hospital mortality than on case-fatality [10]. Furthermore, time delay between the AMI event and hospitalization is more prevalent in low SES subjects, which could contribute to the SES gradient in pre-hospital mortality [11]. Case-fatality is also probably less subjected to SES differences, because The Netherlands is an equity-oriented country with a well developed social system. Every Dutch citizen is obligatory insured with a health insurance company. For citizens with a low income, the government contributes financially. Emergency hospital care is funded by the insurance company without additional costs. Thus, the availability of emergency hospital care should not vary by SES. Therefore, we expect the SES gradient for case-fatality to be smaller than for pre-hospital mortality. However, earlier studies in countries with similar health care systems still have shown a relation between low SES and increased case-fatality after an AMI [8, 12].

Studies on age- and sex-specific relations between SES and short-term AMI mortality [6–8, 13–17] are often restricted to patients younger than 75 years of age, probably due to a lack of large datasets that are needed for this kind of stratification. In the few studies that assessed this relation among patients over 75 years [13–15] it diminished in the elderly. This could be explained by ‘selective survival’ [18]. Selective survival prevents the sicker individuals in low income groups to reach high ages, whereas mortality in the high income groups is postponed to higher ages. This results in comparable mortality risks in the elderly low and high income groups.

With an increasing ageing population in Europe, it is pertinent to gain more insight in SES inequalities in health in the elderly population. We therefore assessed whether the previously observed relationships between household SES and short-term mortality in the younger age groups are also observed in the elderly (i.e. ≥75 years), and whether these relationships vary by sex.

## Methods

### Cohort enrolment

Data were obtained from Dutch national registers; e.g. Hospital Discharge Register (HDR), Dutch Population Register (PR), Cause of Death Register (CDR), and Regional Income Survey (RIO). The registers were used to obtain the following information: the HDR for AMI hospitalizations and co-morbidities, the PR for demographic factors, the CDR for fatal AMI events, and the RIO for income data used as SES indicator. The registers have been described in detail previously [19, 20].

By linking previous registers two cohorts were built. First, a cohort of all Dutch subjects with a first (non-fatal or fatal) AMI event (ICD-9 code 410 and ICD-10 code I21) between January 1st 1998 and December 31st 2007 was constructed. Subjects with a previous hospitalization for AMI from 1995 onwards were excluded. This resulted in a cohort of 260,920 first AMI patients. Second, a cohort of all Dutch patients with a first AMI hospitalization (ICD-9 code 410) between January 1st 1998 and December 31st 2007 was constructed. Subjects with a previous admission for AMI between 1995 and 1997 were excluded. This resulted in a cohort of 199,096 subjects with a first AMI hospitalization.

The first cohort includes all subjects with a first AMI event and was used to study the pre-hospital mortality, defined as death after a first AMI event before hospitalization for this event. The second cohort only includes subjects with a first hospitalization for an AMI event and was used to study the 28-day case-fatality, defined as death within 28 days after the first AMI hospitalization.

### Exposure measures

#### Socioeconomic status

Income data were obtained from the RIO [21]. The RIO started in 1994, when a representative sample of 1.9 million Dutch citizens was selected. Every year, the sample was corrected for emigration and mortality on one hand, and immigration and birth on the other hand. All persons belonging to the households of the sample population (about 5 million) were included in the RIO. SES was defined as the standardized disposable income on household level (adjusted for number of household members) in the year preceding the AMI.

#### Co-morbidity

The presence and the extent of co-morbidity were determined with the Charlson Index Score, which proved to be a reliable and valid method to measure co-morbidity in clinical research [22]. The Charlson Index was constructed using 17 discharge diagnosis of previous admissions, which have been selected and weighted on the basis of the strength of their relation with mortality. The sum of weights represents the Charlson Index Score [23].

### Outcome measures

Pre-hospital mortality, defined as dying after a first AMI event without hospitalization, was determined among all subjects with a first AMI during the study period 1998–2007. Case-fatality, defined as dying within 28 days after a first AMI hospitalization, was determined among subjects with a first AMI hospitalization only.

### Data analysis

To take the effect of inflation and small differences in definition of disposable income over the years into account, the income of the year-specific AMI subjects was divided into quintiles. Subsequently, all year-specific income quintiles were combined in one variable. Baseline characteristics were calculated for every SES quintile of first AMI subjects. Absolute mortality risks were calculated for every SES quintile, stratified by gender and age (<55, 55–64, 65–74, 75–84, ≥85 years). To correct for an unequal age distribution over quintiles, absolute mortality rates were standardized to the age distribution of the study population. Odds Ratio’s (OR) with accompanying 95 % Confidence Intervals (95 % CI), expressing the relation between SES and both short-term mortality outcomes, were calculated using multivariate logistic regression models. These analyses were performed in two ways: first with SES quintile dummy variables, with the highest income quintile (quintile 1) as reference, and second, using SES quintiles as a continuous variable to assess the trends across SES quintiles. Similar models were used for analyses in men and women separately. The same approach was used for analyses in various age strata (<55, 55–64, 65–74, 75–84, ≥85 years). Adjustments were made for potential confounding variables (age, sex, ethnic origin, marital status, degree of urbanization and Charlson Index). Data were analyzed with SPSS software, version 14.0 (SPSS Inc, Chicago, Illinois, USA). All analyses were performed in accordance with privacy legislation Netherlands [24].

## Results

### Baseline characteristics

Of the 260,920 subjects with a first AMI event, 76,351 had income data available in the year preceding their AMI and were included in the study. Patients included in our study were more often male (68.4 vs. 61.6 %), more often married or living together (73.4 vs. 62.2 %), more often living in rural areas (38.4 vs. 36.1 %), had less often co-morbidities (14.9 vs. 16.5 %) and were younger (66.6 vs. 70.1 year) than those not included in our study. Compared to first AMI subjects with a high SES, first AMI subjects with a low SES were older, more often living alone, more often female, more often of non-ethnic Dutch origin, more commonly living in strong urban areas, and had more often co-morbidities (Table 1).

**Table 1:** Characteristics of first AMI subjects with income data available in year prior to the AMI in The Netherlands between 1998 and 2007

	Total	Quintile 1	Quintile 2	Quintile 3	Quintile 4	Quintile 5
Number of subjects	76,351	15,264	15,274	15,272	15,274	15,267
Male (%)	68.4	76.1	72.6	69.6	64.0	59.4
Ethnic Dutch^a^ (%)	87.3	88.0	87.5	87.7	89.6	83.8
Married or living together (%)	73.4	77.6	77.3	76.3	74.3	61.6
Degree of urbanization^b^ (% of quintile)						
Very urban	16.8	15.2	15.6	16.9	17.1	19.1
Urban	23.9	23.1	24.0	24.8	24.1	23.5
Urban/rural	20.9	22.2	21.2	21.4	20.6	19.4
Rural	22.6	24.1	23.6	21.9	21.9	21.4
Very rural	15.8	15.5	15.6	14.9	16.2	16.6
Charson Index >0 (%)	14.9	11.3	12.4	15.1	18.1	17.4
Age at AMI (mean in years)	66.6	63.6	63.6	66.0	70.5	69.2
Standardized disposable income in year preceding AMI (mean in euros)	18,323	31,616	20,749	16,429	13,315	9,508

^a^Both parents born in The Netherlands

^b^Population density (number of residents per km^2^). Very urban = >2,000, urban = 1,001–2,000, urban/rural = 501–1,000, rural = 251–500, very rural = <251

### Pre-hospital mortality

Of all subjects who suffered a first AMI, 15,853 (20.8 %) subjects died outside the hospital (60.5 % men and 39.5 % women). Although the absolute number of total first AMI events was substantially lower in ≥75-year olds compared to those <75 (24,719 and 51,632, respectively), the absolute number of pre-hospital mortality was higher (8,655 and 7,203, respectively) (Table 2). This makes the pre-hospital mortality risk in AMI subjects of ≥75-year old about 2.5 times as high as this risk in AMI patients <75 years. Results of logistic regression showed an inverse relation between SES and pre-hospital mortality, restricted to those with the lowest SES (men: OR = 1.24; 1.15–1.33, women: OR = 1.26; 1.14–1.39). The results did not meaningfully change after correcting for degree of urbanization, ethnic origin, marital status and co-morbidity. After age stratification, the relations in the younger age categories (<75 years of age) persisted in the elderly (75–84 year-old men: OR = 1.26; 1.09–1.47, ≥85 year-old men: OR = 1.26; 1.00–1.58, ≥85 year-old women: OR = 1.20; 0.99–1.46) (Table 3).

**Table 2:** First AMI subjects in every SES-age-sex group (N) and pre-hospital mortality risk (%) in The Netherlands between 1998 and 2007

	<55 N (%)	55–64 N (%)	65–74 N (%)	75–84 N (%)	≥85 N (%)	Total N (%)	ASR
Total							
Total	16,695 (9.3)	16,528 (12.8)	18,409 (19.2)	17,393 (28,7)	7,326 (50.0)	76,351 (20.8)	20.7
Quintile 1^a^	4,147 (9.3)	4,426 (12.1)	3,138 (18.0)	2,483 (28.1)	1,070 (49.2)	15,264 (17.7)	20.1
Quintile 2	4,287 (9.0)	3,888 (10.6)	3,548 (18.0)	2,616 (26.9)	935 (46.8)	15,274 (16.9)	19.2
Quintile 3	3,417 (8.4)	3,201 (12.6)	4,114 (17.7)	3,450 (26.8)	1,090 (46.9)	15,272 (18.7)	19.4
Quintile 4	2,005 (9.1)	2,153 (14.1)	4,495 (20.2)	4,902 (27.7)	1,719 (45.4)	15,274 (23.1)	20.6
Quintile 5	2,839 (11.1)	2,860 (16.2)	3,114 (22.4)	3,942 (33.0)	2,512 (55.9)	15,267 (27.4)	24.2
Men							
Total	13,191 (9.3)	13,166 (12.8)	12,837 (19.4)	10,067 (28.1)	2,957 (45.9)	52,218 (18.4)	18.4
Quintile 1^a^	3,363 (9.4)	3,777 (12.3)	2,394 (18.3)	1,571 (26.7)	505 (45.7)	11,610 (16.1)	17.7
Quintile 2	3,385 (9.5)	3,167 (11.1)	2,566 (18.0)	1,578 (26.4)	394 (42.6)	11.090 (15.5)	17.2
Quintile 3	2,710 (8.4)	2,494 (12.7)	2,910 (17.9)	2,094 (26.1)	473 (41.2)	10,681 (16.9)	17.1
Quintile 4	1,542 (8.5)	1,598 (14.0)	3,025 (21.0)	2,865 (28.5)	746 (43.4)	9,776 (21.8)	18.8
Quintile 5	2,191 (10.6)	2,130 (15.2)	1,942 (22.4)	1.959 (32.2)	839 (52.4)	9,061 (22.7)	21.2
Women							
Total	3,504 (9.2)	3,362 (12.9)	5,572 (18.8)	7,326 (29.5)	4,369 (52.7)	24,133 (26.0)	26.0
Quintile 1^a^	784 (8.5)	649 (10.6)	744 (16.9)	912 (30.6)	565 (52.2)	3,654 (22.9)	25.4
Quintile 2	902 (7.1)	721 (8.3)	982 (18.0)	1,038 (27.7)	541 (49.9)	4,184 (20.5)	23.8
Quintile 3	707 (8.2)	707 (12.2)	1,204 (17.4)	1,356 (27.8)	617 (51.2)	4,591 (22.8)	24.6
Quintile 4	463 (11.2)	555 (14.6)	1,470 (18.5)	2,037 (26.7)	973 (46.9)	5,498 (25.5)	24.5
Quintile 5	648 (12.7)	730 (18.9)	1,172 (22.4)	1,983 (33.8)	1,673 (57.7)	6,206 (34.1)	30.4

*ASR* age standardized rates, standardized to the age distribution of the total study population

^a^Highest income group

**Table 3:** Adjusted^a^ age- and sex-specific odds ratios between SES and pre-hospital mortality after a first AMI in The Netherlands between 1998 and 2007

	Total OR (95 % CI)	<55 OR (95 % CI)	55–64 OR (95 % CI)	65–74 OR (95 % CI)	75–84 OR (95 % CI)	≥85 OR (95 % CI)
Total						
Quintile 1^b^ (reference)	1	1	1	1	1	1
Quintile 2	0.94 (0.88–1.00)	0.97 (0.84–1.13)	0.85 (0.74–0.98)	1.01 (0.89–1.14)	0.94 (0.83–1.07)	0.92 (0.77–1.10)
Quintile 3	0.94 (0.89–1.00)	0.92 (0.78–1.08)	1.01 (0.88–1.16)	0.97 (0.86–1.09)	0.95 (0.84–1.06)	0.92 (0.78–1.10)
Quintile 4	1.01 (0.95–1.07)	0.99 (0.82–1.19)	1.15 (0.98–1.34)	1.14 (1.02–1.29)*	0.98 (0.88–1.09)	0.88 (0.75–1.02)
Quintile 5	1.25 (1.18–1.33)*	1.20 (1.03–1.41)*	1.29 (1.12–1.48)*	1.23 (1.08–1.39)*	1.20 (1.08–1.34)*	1.22 (1.05–1.41)*
*P* trend	<0.001	0.023	<0.001	<0.001	<0.001	<0.001
Men						
Quintile 1^b^ (reference)	1	1	1	1	1	1
Quintile 2	0.96 (0.89–1.03)	1.01 (0.86–1.19)	0.88 (0.76–1.02)	0.99 (0.85–1.14)	0.99 (0.85–1.17)	0.88 (0.67–1.16)
Quintile 3	0.95 (0.88–1.02)	0.92 (0.77–1.10)	0.99 (0.85–1.15)	0.96 (0.83–1.10)	0.99 (0.85–1.14)	0.86 (0.66–1.11)
Quintile 4	1.08 (1.00–1.16)	0.91 (0.73–1.13)	1.09 (0.91–1.29)	1.17 (1.02–1.35)*	1.10 (0.96–1.27)	0.95 (0.76–1.20)
Quintile 5	1.24 (1.15–1.33)*	1.14 (0.95–1.37)	1.18 (1.01–1.37)*	1.21 (1.04–1.41)*	1.26 (1.09–1.47)*	1.26 (1.00–1.58)
*P* trend	<0.001	0.191	0.009	0.001	0.001	0.005
Women						
Quintile 1^b^ (reference)	1	1	1	1	1	1
Quintile 2	0.90 (0.80–1.00)	0.82 (0.57–1.17)	0.75 (0.52–1.08)	1.06 (0.83–1.37)	0.87 (0.71–1.05)	0.94 (0.74–1.19)
Quintile 3	0.93 (0.84–1.04)	0.94 (0.65–1.35)	1.15 (0.82–1.62)	1.00 (0.78–1.28)	0.88 (0.73–1.06)	0.97 (0.77–1.22)
Quintile 4	0.90 (0.81–1.00)	1.31 (0.89–1.93)	1.40 (0.99–1.98)	1.09 (0.86–1.37)	0.82 (0.69–0.97)*	0.82 (0.66–1.01)
Quintile 5	1.26 (1.14–1.39)*	1.48 (1.04–2.09)*	1.81 (1.31–2.49)*	1.29 (1.02–1.64)*	1.10 (0.93–1.31)	1.20 (0.99–1.46)
*P* trend	<0.001	0.007	0.000	0.109	<0.001	<0.001

^a^Adjusted for age, ethnic origin, marital status, degree of urbanization, Charlson Index. In unstratified analyses also adjusted for sex

^b^Highest income group

* *P* < 0.05

### Case-fatality

Of all subjects who suffered a first AMI, 60,498 (79.2 %) were hospitalized (70.5 % men and 29.5 % women); 9,656 (16.0 %) of them died within 28 days (62.5 % men and 37.5 % women). Although the absolute number of total AMI hospitalizations was substantially lower in ≥75-year olds compared to those <75 years (16,071 and 44,427, respectively), the absolute number of case-fatality was higher (5,227 and 4,423, respectively) (Table 4). This makes the case-fatality risk in the AMI patients of ≥75-year olds more than 3 times as high as this risk in AMI patients <75 years. Results of logistic regression showed a gradual increase in case-fatality risk with decreasing SES, but only in men (lowest vs. highest SES: OR = 1.28; 1.17–1.41). The results did not meaningfully change after correcting for degree of urbanization, ethnic origin, marital status and co-morbidity. After age stratification, a consistent inverse relation in men of 55 years of age and above was shown, persisting in the older age categories (75–84 years: OR = 1.26; 1.06–1.50, ≥85 years: OR = 1.36; 0.99–1.85). In women, no clear SES gradients in case-fatality were found (Table 5).

**Table 4:** First hospitalized AMI patients in every SES-age-sex group (N) and case-fatality risk (%) in The Netherlands between 1998 and 2007

	<55 N (%)	55–64 N (%)	65–74 N (%)	75–84 N (%)	≥85 N (%)	Total N (%)	ASR
Total							
Total	15,144 (5.2)	14,412 (8.3)	14,871 (16.4)	12,405 (28.9)	3,666 (44.8)	60,498 (16.0)	16.0
Quintile 1^a^	3,597 (4.9)	3,781 (7.3)	2,462 (15.0)	1,730 (27.1)	526 (43.7)	12,096 (12.6)	14.9
Quintile 2	3,740 (5.3)	3,322 (8.1)	2,761 (16.0)	1,809 (28.7)	469 (45.8)	12,101 (13.6)	15.9
Quintile 3	3,187 (5.6)	2,789 (8.8)	3,265 (15.7)	2,318 (26.7)	542 (44.6)	12,101 (14.9)	15.6
Quintile 4	1.959 (4.6)	2,000 (9.1)	3,669 (16.2)	3,560 (28.9)	914 (42.7)	12,102 (18.9)	15.8
Quintile 5	2,661 (5.1)	2,520 (9.0)	2,714 (19.2)	2,988 (31.9)	1,215 (46.6)	12,098 (19.9)	17.5
Men							
Total	11,693 (4.7)	11,484 (7.9)	10,345 (16.4)	7,237 (29.4)	1,599 (46.6)	42,628 (14.2)	14.2
Quintile 1^a^	2,391 (4.6)	3,223 (6.9)	1,875 (14.2)	1,115 (27.9)	265 (43.8)	9,409 (11.2)	13.0
Quintile 2	2,921 (4.8)	2,692 (7.5)	2,005 (15.8)	1,095 (29.3)	210 (50.0)	8,923 (12.2)	14.1
Quintile 3	2,530 (5.0)	2,191 (8.4)	2,323 (15.8)	1,427 (26.3)	264 (44.3)	8,735 (13.4)	13.7
Quintile 4	1,526 (4.1)	1,485 (8.9)	2,432 (16.9)	2,077 (29.5)	415 (44.1)	7,935 (17.7)	14.3
Quintile 5	2,055 (4.5)	1,893 (8.8)	1,710 (19.7)	1,523 (33.2)	445 (50.3)	7,626 (17.4)	16.0
Women							
Total	3,181 (7.0)	2,928 (10.0)	4,526 (16.4)	5,168 (28.3)	2,067 (43.4)	17,870 (20.3)	20.2
Quintile 1^a^	666 (6.2)	558 (9.5)	587 (17.5)	615 (25.7)	261 (43.7)	2,687 (17.5)	19.6
Quintile 2	819 (7.1)	630 (10.3)	756 (16.8)	714 (27.9)	259 (42.5)	3,178 (17.6)	20.2
Quintile 3	657 (7.8)	598 (10.4)	942 (15.5)	891 (27.3)	278 (45.0)	3,366 (18.6)	20.1
Quintile 4	433 (6.5)	515 (9.7)	1,237 (15.0)	1,483 (28.1)	499 (41.5)	4,167 (21.3)	19.5
Quintile 5	606 (7.4)	627 (9.9)	1,004 (18.2)	1,465 (30.5)	770 (44.4)	4,472 (24.1)	21.5

*ASR* age standardized rates, standardized to the age distribution of the total study population

^a^Highest income group

**Table 5:** Adjusted^a^ age- and sex-specific odds ratios between SES and case-fatality after a first AMI hospitalization in The Netherlands between 1998 and 2007

	Total OR (95 % CI)	<55 OR (95 % CI)	55–64 OR (95 % CI)	65–74 OR (95 % CI)	75–84 OR (95 % CI)	≥85 OR (95 % CI)
Total						
Quintile 1^b^ (reference)	1	1	1	1	1	1
Quintile 2	1.09 (1.01–1.18)*	1.09 (0.88–1.34)	1.08 (0.91–1.29)	1.08 (0.93–1.25)	1.09 (0.94–1.26)	1.10 (0.86–1.42)
Quintile 3	1.06 (0.98–1.14)	1.17 (0.95–1.46)	1.15 (0.96–1.38)	1.03 (0.89–1.19)	0.99 (0.86–1.14)	1.05 (0.83–1.35)
Quintile 4	1.09 (1.01–1.17)*	0.98 (0.75–1.27)	1.16 (0.95–1.42)	1.06 (0.92–1.23)	1.10 (0.96–1.25)	0.99 (0.79–1.23)
Quintile 5	1.22 (1.13–1.32)*	1.07 (0.85–1.35)	1.13 (0.94–1.37)	1.27 (1.09–1.47)*	1.24 (1.08–1.41)*	1.15 (0.93–1.41)
*P* trend	<0.001	0.565	0.489	0.009	0.002	0.481
Men						
Quintile 1^b^ (reference)	1	1	1	1	1	1
Quintile 2	1.10 (1.00–1.21)	1.06 (0.83–1.35)	1.07 (0.88–1.31)	1.13 (0.94–1.34)	1.08 (0.89–1.30)	1.30 (0.90–1.88)
Quintile 3	1.06 (0.97–1.17)	1.13 (0.88–1.46)	1.18 (0.96–1.44)	1.10 (0.93–1.31)	0.93 (0.78–1.11)	1.06 (0.75–1.51)
Quintile 4	1.13 (1.03–1.24)*	0.94 (0.69–1.29)	1.23 (0.89–1.55)	1.19 (1.00–1.41)	1.09 (0.93–1.28)	1.07 (0.78–1.47)
Quintile 5	1.28 (1.17–1.41)*	0.98 (0.75–1.30)	1.19 (0.97–1.48)	1.40 (1.17–1.68)*	1.26 (1.06–1.50)*	1.36 (0.99–1.85)
*P* trend	<0.001	0.742	0.293	0.003	0.003	0.216
Women						
Quintile 1^b^ (reference)	1	1	1	1	1	1
Quintile 2	1.05 (0.91–1.21)	1.20 (0.79–1.82)	1.09 (0.74–1.59)	0.94 (0.70–1.25)	1.12 (0.87–1.42)	0.95 (0.67–1.35)
Quintile 3	1.02 (0.89–1.17)	1.31 (0.86–2.02)	1.05 (0.71–1.55)	0.84 (0.64–1.11)	1.09 (0.86–1.38)	1.05 (0.74–1.48)
Quintile 4	0.98 (0.86–1.11)	1.11 (0.67–1.83)	0.97 (0.64–1.46)	0.80 (0.61–1.04)	1.12 (0.90–1.39)	0.92 (0.68–1.25)
Quintile 5	1.11 (0.98–1.26)	1.35 (0.87–2.12)	0.96 (0.65–1.42)	0.98 (0.75–1.29)	1.23 (0.99–1.52)	1.02 (0.76–1.35)
*P* trend	0.172	0.668	0.963	0.290	0.400	0.901

^a^Adjusted for age, ethnic origin, marital status, degree of urbanization, Charlson Index. In unstratified analyses also adjusted for sex

^b^Highest income group

* *P* < 0.05

## Discussion

Previous studies already showed a SES gradient in short-term mortality after AMI below 75 years of age. We expand this evidence by showing that this relationship persists in 75 year-olds and beyond. Most previous studies excluded AMI patients ≥75 years of age. Those who included them often found less pronounced relations in the elderly [13–15]. The lack of a socioeconomic gradient among the elderly is often explained by ‘selective survival’ [18], which prevents the sicker individuals in low income groups to reach high ages. Subsequently, elderly low income subjects are healthier than their younger counterparts, and SES gradients diminish with age. In our study, the elderly low SES AMI subjects still had a higher short-term mortality compared to the elderly high SES AMI subjects. So even in case of selective survival, relations did not disappear. Besides selective survival, misclassification of SES may have influenced the diminishing relation with age in previous studies. The SES indicators in those studies were on neighborhood level instead of individual or household level. Neighborhood level SES is more sensitive to misclassification and underestimation of results, especially in elderly living in institutional care where neighborhood SES is probably less similar to individual SES. The results in our study are based on household level SES, which is a more reliable indicator of a persons true SES and consequently less prone to misclassification [25].

As in many European countries the Dutch population is increasingly ageing, which is partly caused by an increase in life expectancy. Moreover, the baby boomers born close after the Second World War are reaching the age of 65 by now and within ten years they will belong to the group of ≥75 year olds. Our study showed that short-term mortality after a first AMI is about 3 times as high in ≥75 year olds compared to <75 year-olds. Diminishing SES differentials in short-term mortality in the elderly population might thus be a viable step in improving population health, and consequently reduce health care costs. Interventions intended to promote healthy lifestyle should not only focus on direct change but also on maintenance of change. Elderly persons must be included in these interventions, because some lifestyle changes, like smoking cessation, [26] still have beneficial health effects at an older age. Additionally, physicians need to be more vigilant on the elderly population from a low SES background, for example regarding therapy education and compliance.

### Pre-hospital mortality

Our results showed an increased risk of dying immediately after a first AMI event in patients with the lowest SES, which was of the same magnitude in men and women. The relations were only present when comparing the two most deviating SES quintiles, while previous studies reported graded relations over SES groups [7, 8, 17]. This implies that in The Netherlands only the least wealthy group has a disadvantage with respect to pre-hospital mortality risk after AMI. There are several possible explanations for this, including more unfavorable risk factor profiles [9] and seeking medical care too late in low SES subjects [11]. Also larger and more severe AMI in low SES groups [27] and differences in medical care prior to the AMI could have influenced pre-hospital mortality risk adversely.

### Case-fatality

We expected that the SES-gradients in case-fatality would be less pronounced compared to the SES gradients in pre-hospital mortality. This was only correct for women, where no clear relation with case-fatality was found. Our results still showed an inverse relation between SES and case-fatality in men, which was of a similar magnitude as the one with pre-hospital mortality. This is in line with some previous studies [8, 17, 28] using income on an individual level as SES indicator. The factors involved in an increased pre-hospital mortality risk in low SES groups mentioned before (unfavorable risk factor profiles, seeking medical care too late, and larger and more severe infarcts) can proceed after hospitalization in the survivors, and increase the case-fatality risk. More co-morbidity (e.g. diabetes) [29] and differences in aggressiveness of treatment after AMI [30] are other possible explanations of the SES gradient in case-fatality in men.

A SES gradient in case-fatality was found in men but not in women. Salomaa et al. [8] presented some possible explanations for this sex difference in Finland, which has a similar health care system as The Netherlands. Their research showed a delay from onset of symptoms to medical presence in men with a low SES compared to men with a high SES. In women, no SES difference was found. This time delay in hospitalization could postpone necessary treatment in low SES men, which may increase case-fatality. They also found that angiography within 28 days after hospitalization was significantly more often performed in men with high SES than in men with low SES. This was not the case in women. Delay in angiographic procedures could mask the severity of the AMI and might withhold the patient from necessary treatments. This implies that even in an equity-oriented country with a well developed social system, SES gradients in health care exist.

### Limitations

There are some limitations that should be mentioned. Firstly, although there has been corrected for co-existing diseases, some of them (e.g. diabetes) have been underreported in the HDR. Since persons with a low SES have more co-morbidity, the relations reported in this study may have been overestimated. Secondly, the fact that we had no information regarding previous admissions before 1995 might have resulted in some ‘first-time’ AMI events that were actually recurrent events. Recurrence of AMI is non-significantly more common in low SES groups than in high SES groups. Therefore, the inclusion of recurrent events might have led to overestimation of mortality risks (as recurrent events are usually more severe) especially in the low SES groups. Thirdly, we were not able to go into depth concerning underlying mechanisms of our findings, due to the absence of information regarding risk factors, event severity, procedures and medication use. Finally, AMI subjects included in our study (subjects with income data available) had more favorable characteristics compared to AMI subjects not included in our study, which are in general related to better health and a higher income. Including all first AMI patients of the entire Dutch population would probably lead to a larger spread in income range, resulting in more pronounced relations.

### Strengths

By using national registers we were able to build a cohort of 260,920 first AMI subjects over a 10 year time span including all age ranges. Previously it has been investigated that the overall quality of Dutch national registers is high [31]. ICD-9 code 410 and ICD-10 codes I21–22 were used to identify patients with an AMI in the Hospital Discharge Register and the Cause of Death Register respectively. The validity of these ICD-codes was previously assessed, resulting in a sensitivity of 84 % and a positive predictive value of 97 %. This indicates that most patients coded with ICD-9 code 410 and ICD-10 codes I21–22 actually experienced an AMI event, and are thus correctly coded [32]. Furthermore, we had the opportunity to link the Regional Income Survey providing income data on household level. Most other studies used surrogate indicators of SES on neighborhood level, which inevitably leads to some misclassification and may bias the results towards the null [25]. Additionally, the high number of AMI subjects in our study gave us the opportunity to stratify by sex and age, while keeping enough power for the analyses. Unlike most previous studies we included elderly patients (≥75 years of age). With the ageing of the Western population, the number of persons at risk for short-term mortality after a first AMI grows simultaneously. This makes it important to include the elderly population when studying this matter. Because pre-hospital mortality and case-fatality partly differ in underlying mechanisms, we studied both outcome measures separately. This large register-based study includes persons of all ages, a SES indicator on household level, stratified analyses by sex and age, and distincts between pre-hospital mortality and case-fatality. As such this study is, apart from very relevant for clinicians and policy makers, unique and expands existing evidence.

## Conclusion

In men there are SES inequalities in both pre-hospital mortality and case-fatality after a first AMI, in women these SES inequalities are only shown in pre-hospital mortality. The inequalities persist in the elderly. Interventions should focus on healthy lifestyle promotion and maintenance over the life course in low SES groups, and should not be restricted to the younger population. Clinicians working in primary as well as secondary care need to be more vigilant on the population with a low SES background, including the elderly.
